# Electromagnetic shielding effectiveness of three-dimensional multilayered interlaced woven fabrics using stainless steel fibers

**DOI:** 10.1016/j.heliyon.2025.e41669

**Published:** 2025-01-04

**Authors:** Brigita Kolcavová Sirková, Veronika Tunáková, Maros Tunák, Karol Jezik

**Affiliations:** a Department of Technologies and Structures, Technical University of Liberec, Czech Republic; b Department of Material Engineering, Technical University of Liberec, Czech Republic; c Department of Textile Evaluation, Technical University of Liberec, Czech Republic

**Keywords:** Three-dimensional, Woven fabric, Weave, Electromagnetic shielding, Interlacing

## Abstract

This study explores and discusses the design, the manufacturing and the morphology of three-dimensional (3D) multilayered weft interlaced woven fabrics using stainless steel fibers on the electromagnetic shielding efficiency (SE). Design solutions of 3D multilayered interlaced fabrics in relation to electromagnetic shielding efficiency are still not sufficiently investigated. Moreover, this study aims to analyze the differences in the internal geometry of 3D multilayered weft interlaced fabrics with different number of layers and frequency of connecting points in multilayered woven fabrics on electromagnetic SE. For this study, the input staple yarn 2 × 20 tex (mixed 80 % polyester/20 % stainless steel fibers) is used for production of approximately 28 types of different 3D multilayer weft interlaced woven fabrics (two and three-layer), with connecting points ranging from 0.5 cm × 0.5 cm–5 cm × 5 cm. The comparison of internal fabric microstructures indicates statistically significant differences in relation to SE, and this contribution extends the theoretical guidance of woven fabric construction with the design and production of special 3D multilayered interlaced fabrics with controlled SE.

## 1 Introduction

Electromagnetic radiation encompasses a broad spectrum of waves, ranging from radio to microwaves, infrared, visible light, ultraviolet, X-rays, and gamma rays. These waves can be emitted from various sources, such as electronic devices, power lines, wireless communication systems, and even natural sources like the sun. While some forms of electromagnetic radiation, such as visible light, are generally harmless, others can cause problems with interferences (radio waves or microwaves) or can be ionizing and pose health risks (X-rays or gamma rays).

Electromagnetic shielding refers to the process of blocking or reducing the penetration of electromagnetic radiation through the use of various materials or structures. Textile structures can be crucial in providing electromagnetic shielding due to their versatility, lightweight nature, deformability, air and water vapor permeability, and ease of integration into different applications.

Textile structures designed for electromagnetic shielding typically contain conductive components (metal fiber/wire, metal coating, conductive polymer coating, carbon fiber, etc.) that can reflect or absorb electromagnetic radiation [1]. The primary mechanism of shielding is usually through reflection.

Textile structures for electromagnetic shielding can take various forms, such as fabrics, garments, laminates, or composites. Conductive textiles can be woven, knitted, non-woven, or printed onto traditional fabrics. Woven fabrics offer several advantages for electromagnetic shielding compared to knitted or non-woven fabrics: higher electromagnetic shielding effectiveness (SE) due to regular and tightly interlaced structure; enhanced stability providing that the shielding ability remains consistent even under varying environmental conditions or mechanical stress; improved durability (better tensile strength, more resistant to tearing and abrasion) ensuring that the electromagnetic shielding properties of the fabric remain intact over extended periods of use.

The woven fabrics are divided into two-dimensional (2D) and 3D according to the number of thread systems involved in their formation [2]. Regarding the basic classification of the number of thread systems, 2D woven fabrics are simple woven fabrics with one warp and one weft system. With respect to their structure, the essential range of 2D woven fabrics is dimensionally defined by their width and length. The diameter of the ends expresses the third dimension of 2D fabric and picks in cross-section of woven fabric. The basic classification of 3D woven fabrics divides them into three elementary groups: (a) multiple warp woven fabrics – these are fabrics where structure is formed by two or more warp systems and one weft system; (b) multiple weft woven fabrics – these are fabrics where structure is formed by one warp system and two or more weft systems and (c) multiple woven fabrics – these are fabrics where structure is formed by two or more warp systems and two or more weft systems. 3D multiple woven fabric can be made based on the multilayer principle of threads interlacing (including also as layered without a connector and hollow - tubular fabric), the orthogonal principle of threads interlacing, and angle interlock principle [2]. The threads of these warp and weft systems in 3D fabrics are structurally laid in layers on top of each other, creating a third dimension of 3D fabrics. The presented contribution is focused on group c) 3D multi-layered weft interlaced woven fabrics. Several parameters of woven fabrics can affect their electromagnetic shielding effectiveness. Here are some key parameters to consider: (a) electrical conductivity of basic elements: yarns or fibers because the choice of conductive component is a crucial factor [[3], [4], [5], [6], [7], [8], [9], [10]], (b) fabric cover and density associated with the warp and weft sett, whereas higher density enhances the fabric's ability to block electromagnetic radiation [11,12], (c) fabric thickness – thicker fabrics typically provide higher shielding as known from theory [10,11], (d) weave pattern. The weave pattern of the fabric also plays a significant role in determining its electromagnetic shielding effectiveness. Different weave patterns, such as plain, twill, or satin, affect the fabric's density, coverage, and overall structure [[13], [14], [15]].

Moreover, some weave patterns may create a more interconnected conductive network, improving shielding performance. The cover dimensions and joining (seams) are not discussed in more detail here. Nevertheless, these variables also influence the total shielding effectiveness of the shielding enclosure.

Woven fabrics are also the key reinforcement type for the composites' production, whereas stacking woven fabrics one over the other is commonly used [16]. The effect of stacking of single woven fabrics on total electromagnetic shielding is relatively well explored. It is known that unidirectional placement of conductive yarn provides the lowest SE (SE < 10 dB) [17]. By doubling unidirectional conductive yarn structures, SE can be increased up to 40 dB, whereas overlapping with 90° is preferred because a symmetrical, non-interlocked grid-like structure is formed [17]. Even more advantageous is the layering of interlaced yarn, creating a woven structure [4,[17], [18], [19]]. It was confirmed that the more layers, the higher the SE, and this dependence can be described by power function [4]. Nevertheless, delamination of these stack-reinforced composites underperformance represents a significant disadvantage in their use [16].

That is why the sewing and weaving of 3D fabrics are promising technology, which is also supported by the fact that the mechanical properties (tensile, flexural, and impact) of 3D woven fabric reinforced composites were found to be better than those of the 2D reinforced composites [20]. In addition, the increased thickness of 3D woven fabrics and their internal interlocked structure offer more opportunities for developing electromagnetic shielding enclosures with controllable properties [21]. Statistically significant effect of copper filament diameter used as core of electrically conductive yarn, yarn diameter and yarn fineness on SE of 3D orthogonal woven fabrics was proved at [21], and the SE of these 3D fabrics ranged between 18 and 35 dB for frequency 8–12 GHz. Polyamide 3D spacer fabrics were used as a substrate for coating with reduced graphene oxide/carbon nanotubes, and an SE of around 15 dB at a frequency of 2 GHz was achieved [22]. Authors described in the paper [23] the effect of the architecture of the 3D woven reinforcements made of carbon rovings on SE, and they concluded that the highest SE around 55 dB at frequency 12 GHz was achieved by 3D interlock woven fabric/epoxy composite. The authors of the paper [21] found that undulating the conductive yarns through the thickness of the 3D warp interlock woven fabrics positively affects SE.

As mentioned above, the multilayer interlaced fabrics represent one class of 3D multiple fabrics. Electromagnetic shielding characteristics of multilayer 3D conductive fabrics made of cotton/copper wrapped yarns were studied in Ref. [24], and it can be concluded that cell-type spacer fabric had the highest SE (>30 dB at 8 GHz) compared to orthogonal, angle interlock, multi-tubular sper, and tubular contour 3D fabric samples. The degree of buckling of 3D bidirectional angle-interlock woven fabric was studied in Ref. [25]. It was found that the higher the degree of buckling, the higher the SE. According to the authors, the SE also depends on the shape of the yarn cross-section, fabric thickness, and fiber volume fraction. Application of electrically conductive 3D woven fabrics in other electromagnetic wave fields, such as antennas, was reported for example, in Refs. [26,27].

While the effect of selected parameters (more precisely, the content of the conductive component, number of layers and their orientation, warp, and weft sett, fabric pattern) on the electromagnetic shielding ability of fabrics is relatively well described, the fabric structure itself, incl. the influence of the interlacing of individual yarns is still poorly researched, especially for 3D woven fabrics.

This study explores and discusses the morphology of three-dimensional (3D) multi-layered weft interlaced woven fabrics on the resulting electromagnetic shielding efficiency. Design solutions for 3D multi-layered interlaced fabrics concerning electromagnetic shielding efficiency have not been sufficiently investigated. The number of interlacing points and their distribution in the area of the multilayer woven (double-layered, three-layered) fabrics on SE effectiveness will be explored to find out how the interlacing affects both the structure and, subsequently, the shielding barrier ability of an electrically conductive multi-layered woven fabrics. CAD system 3D WEAVE “TECH” was used for the simulation of the fabric structure of 3D multi-layered weft interlaced woven fabrics. Mixed staple yarns 2 × 20 tex (mixed 80 % polyester/20 % stainless steel fibers) were used for the creation of approximately 28 types of different 3D multilayer weft interlaced woven fabrics (double and three-layered), with connecting points ranging from 0.5 cm × 0.5 cm–5 cm × 5 cm. The comparison of internal fabric microstructures indicates statistically significant differences. This contribution extends the theoretical guidance of woven fabric construction with the design and production of unique 3D multi-layered interlaced fabrics with controlled SE.

## 2 Materials and methods

### 2.1 Sample preparation and description of experimental woven fabric parameters

**Fig. 1:**
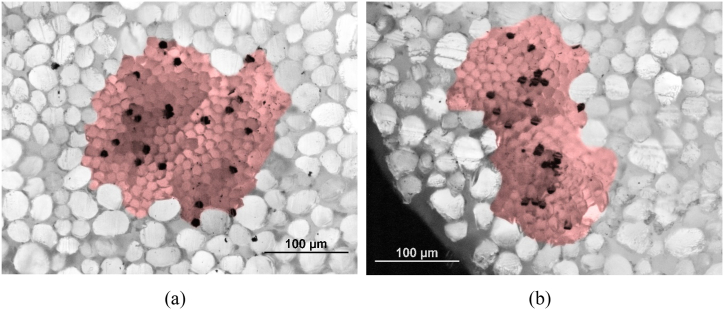
**(a), (b).** Microscopic images of a cross-section of hybrid twisted yarn (displayed in red), stainless steel fibers are black (*note: microscopic image: optical microscope NIKON ECLIPSE LV100, camera NIKON DIGITAL SIGHT DS-5M, with the deep focus technique and 20* × *magnification (0.*17 μm*/px, resolution 2560* × *1920)*).

The automatic sampling rapier weaving machine CCI Evergreen (working width: 520 mm) with electronic jacquard shedding mechanism (BONAS, a total capacity of the hooks: 1348) was used to produce the experimental double-layer and three-layer 3D multi-layered weft interlaced woven fabric samples (*note: as part of the experiment 2D woven fabrics were woven for comparison of behavior of 3D multi-layered weft interlaced woven fabrics. Construction parameters of 2D fabrics are identical to individual layers in 3D multi-layered fabrics*). The jacquard shedding mechanism comprised 1300 hooks. All experimental 2D and 3D woven samples were produced with identical construction parameters in warp and weft. Construction parameters of input material are: twisted yarn with yarn count [tex]: 2 × 20 (Fig. 1), mixed staple 80 % polyester/20 % stainless steel fiber, parameters of polyester fibers: length [mm]: 90; count [dtex]: 1.3; parameters of wire: 316L alloy; diameter [μm]: 6.5; count [dtex]: 3.85; length [mm]: 90; strength [cN]: 5.52; elongation [%]: 1.29. Yarns were purchased from company SINTEX, a. s., Czech Republic and properties of fibers were taken from the product sheet provided by the yarn manufacturer. The choice of material is connected with the application of the designed structure. The study suggests applications of designed textile structures as clothing textiles for protective light wear.

**Fig. 2:**
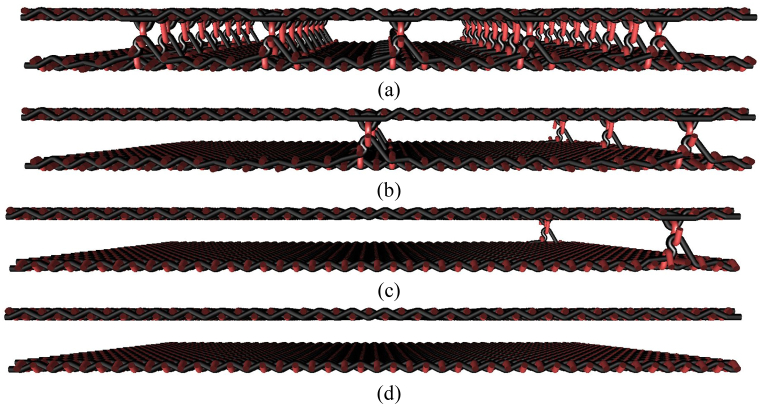
Presentation of selected cross-sections of 3D weft-interlaced woven fabrics - two layers with distribution of connecting points layer-to-layer: (a) 0.5 cm × 0.5 cm, (b) 1.5 cm × 1.5 cm, (c) 3 cm × 3 cm, (d) 2D layered woven fabrics without connecting points.

**Fig. 3:**
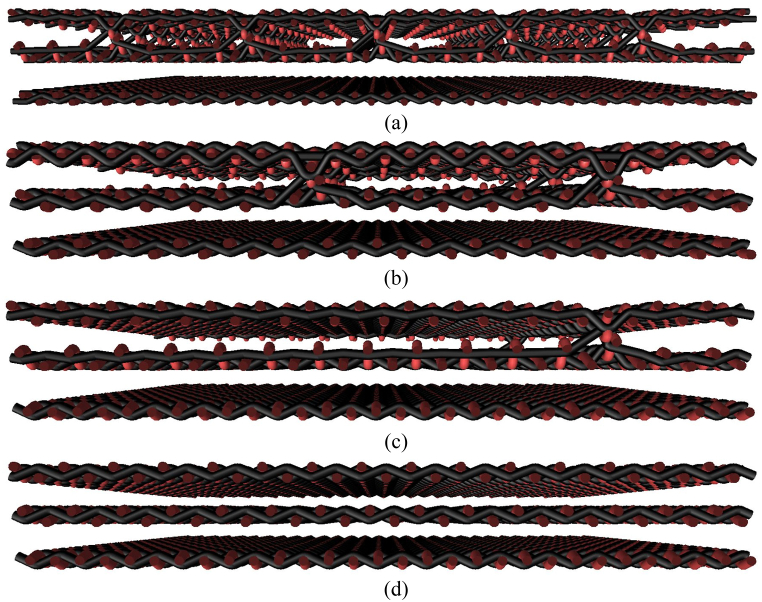
Presentation of selected cross-sections of 3D weft-interlaced woven fabrics - three layers having interlaced only two layers with a distribution of connecting points layer-to-layer: (a) 0.5 cm × 0.5 cm, (b) 1.5 cm × 1.5 cm, (c) 3 cm × 3 cm, (d) 2D layered woven fabrics without connecting points.

**Fig. 4:**
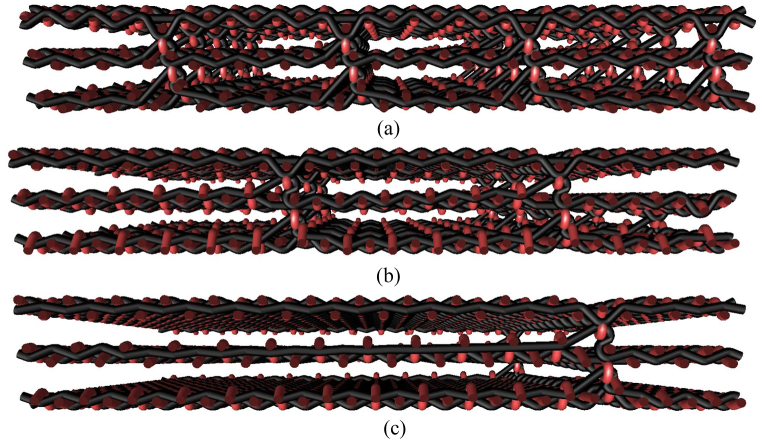
Presentation of selected cross-sections of 3D weft-interlaced woven fabrics - three layers with distribution of connecting points layer-to-layer, (a) 0.5 cm × 0.5 cm, (b) 1.5 cm × 1.5 cm, (c) 3 cm × 3 cm.

Construction parameters of tested woven fabrics are: type of connection point in 3D multi-layered fabric: weft connecting; interlacing of threads in 2D and 3D woven fabrics: plain weave; double-layer 3D woven fabric sett of ends and picks (cm^−1^): 13.5; three-layers 3D woven fabric sett of ends and picks (cm^−1^): 8.8.3D multi-layered interlaced woven fabrics having two layers interlaced were produced using distribution of connecting points ranging from 0.5 cm × 0.5 cm–5 cm × 5 cm (Fig. 2), three layers were interlaced with connecting points ranging from 0.5 cm × 0.5 cm–3 cm × 3 cm, while the three-layer samples were produced in two variants: (a) only two layers are interconnected, one is free (see Fig. 3), (b) all three layers are interconnected layer-to-layer (Fig. 4). The study uses a layer-to-layer technique. The through-layers interlacing variant is not used in the study. The through-layers interlacing creates large structural deformations of the connected area, which leads to the formation of large gaps in the connection area. Such an uneven structure is not suitable for clothing applications.

The basic parameters of experimental 2D and 3D multi-layered interlaced woven samples are presented in Table 1.

**Table 1:** Parameters of experimental 2D fabric and 3D multi-layered weft interlaced woven fabric samples.

	Type of fabric	Sett of ends/picks one layer [/cm]	Number of layers	Number of interlac. layers	Distribution of connect. points [cm]	Number of connect. points [/100 cm^2^]	CF [%]	Thickness [mm]	Fabric weight [g/m^2^]
Group 0	2D	8.8/8.8	1	–	–	–	50.71	0.36	87.8
	2D	13.2/13.2	1	–	–	–	81.02	0.41	131.7
Group 1	3D	13.2/10	2	0	0	0	97.93	0.77	237.8
				2	0.5	400	95.37	0.73	251.4
		13.2/13.2	2	0	0	0	99.69	0.77	264.4
				2	0.36	555	97.38	0.73	277.6
		13.2/18	2	0	0	0	99.94	0.81	294.4
				2	0.28	714	99.52	0.80	300.1
		13.2/24	2	0	0	0	99.99	0.88	343.2
				2	0.21	952	99.85	0.84	354.4
Group 2	3D	13.2/13.2	2	0	0	0	98.43	0.81	264.4
				2	0.5	400	97.38	0.79	265.2
					1	100	95.32	0.80	267.7
					1.5	44.44	95.78	0.79	267.4
					2	25	96.93	0.80	266.9
					2.5	16	95.84	0.80	266.3
					3	11.11	96.42	0.80	265.7
					4	6.25	97.95	0.80	264.9
					5	4	96.78	0.79	265.4
Group 3	3D	8.8/8.8	2	0	0	0	82.11	0.68	175.6
				2	0.5	400	88.75	0.70	176.7
					1.5	44.44	87.15		175.3
					3	11.11	87.16		175.9
Group 4	3D	8.8/8.8	3	0	0	0	94.7	1.02	263.4
				3	0.5	800	94.22	1.00	265.4
					1.5	88.88	92.35	1.06	264.9
					3	22.22	94.56	1.08	263.9
Group 5	3D	8.8/8.8	3	2	0.5	400	97.06	1.05	264.8
					1.5	44.44	95.62		263.6
					3	11.11	95.99		264.3

**Fig. 5:**
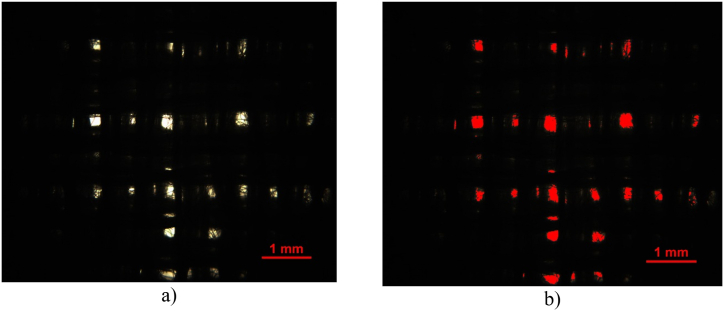
Presentation of scanning and evaluating the CF of woven fabric a) the captured input image of 2-layer 3D woven fabric with connecting points 0.5 cm × 0.5 cm b) binary images of covered area of yarn in woven fabric (black colour, red is pores) for experimental determination of CF of woven fabric.

The woven fabric thickness, area coverage Cover Factor (CF), and woven fabric weight shown in Table 1 are measured in this study for a more objective perspective of the influence of 2D and 3D multi-layered interlaced woven fabrics relative to SE. Experimental fabric thickness is defined as the perpendicular distance between two reference plates exerting a pressure of 1 kPa or less on the textile. The thickness of the woven fabric is given by standard EN 5084 [28]. The internal standard gives the methodology for evaluating the CF of woven fabric IS 23-107-01/01 [29]. The methods of scanning and evaluating the CF of woven fabric consist of determining the yarn covered area of woven fabric microscopic specimen in transmitted light, see Fig. 5. Finaly CF is given as the ratio of the areas: the yarns covered area (black colour, see Fig. 5b) to the total scanned area of the image (the entire area of the presented image Fig. 5b). Images of woven fabrics were successfully observed and captured by an optical microscope NIKON ECLIPSE E 200, camera NOPTIK PROGRES CT3, using a 2 × 0.6 magnification (2.65μm/px, resolution 2048 × 1536). The evaluation of individual images of woven fabric and preparation of binary images of woven fabric specimens was performed using image analysis software, NIS-Elements. Measurements was repeated 50 times for each sample.

### 2.2 Methodology of measuring the electromagnetic SE of 3D multi-layered weft-interlaced woven fabric samples

The ASTM D 4935–10 [30] standard was chosen to evaluate the electromagnetic barrier capability of 2D and 3D multi-layered weft interlaced woven samples. This standard is currently one of the most used, especially for evaluating fiber-based fabrics [[29], [30], [31], [32]]. The set-up consisted of a Rhode&Schwarz ZNC3 vector analyzer together with an Electro-Metrics Corp. EM-2107A coaxial specimen holder. This test fixture is an enlarged section of the coaxial transmission line. It complies fully with the requirements of ASTM test method D4935-10 for evaluation of the electromagnetic shielding effectiveness of planar material due to a plane wave (far field EM wave). The advantages offered by this test technique are the relatively small random uncertainty (which is ±1 dB), the necessity of a relatively small test sample (15 cm dia disk) and the possibility to determine the components of the total electromagnetic shielding effectiveness (reflected, absorbed). The measurement method is valid over a frequency range of 30 MHz to 1.5 GHz. In contrast, in this experiment, more attention will be given to the highest frequency in the measured range, which is *f* = 1.5 GHz, because they are close to the transmission frequency of various devices such as mobile phones (e.g., GSM 1800), wireless LAN systems (e.g., 2.4 GHz), radars (e.g., L band 1–2 GHz), and GPSs (L1 Band: 1575.42 MHz). The samples were air-conditioned before testing (*T* = 23 ± 3 °C, *RH* = 50 ± 10 %), and the measurements were performed at five randomly chosen sample locations (*n* = 5) to facilitate the subsequent statistical analysis.

Before calculating basic statistical characteristics (central tendency, dispersion) and using ANOVA, the sample data were tested for homogeneity. Lilliefors test [34] was used to prove that data comes from distribution in the normal family, against the alternative that it does not with the significance level α = 0.05. It was confirmed that for variables: SE, thickness and CF test result was *h* = 0 to indicate that the test doesn't reject the null hypothesis. The Bartlett test [33] was used to test whether multiple samples are from populations with equal variances. The *p*-value >0.0.05 indicates that the test doesn't reject the null hypothesis (at significance level α = 0.05) that the variances are equal across different sample groups, especially variable: thickness and cover factor depending on different numbers of connecting points.

## 3 Results and discussion

This section investigates the differences in the internal geometry of 3D multi-layered weft interlaced fabrics with different layers and frequencies of connecting points in multi-layered woven fabrics on the electromagnetic SE. For a closer examination of this effect, two-layer and three-layer 3D multi-layered weft interlaced fabrics layer-to-layer connection, and three-layer sample having connecting points in 2 layers only were woven and analysed. Individual findings are supported by investigating the change in the internal structure of the 3D samples, which is caused by the interlacing of layers. For this study, the identical input yarn (made from a mixture of traditional and extremely thin discrete stainless steel fibers) is used in warp and weft systems for the production of approximately 28 types of different 3D multilayer weft-interlaced woven fabrics (two and three layers), with connecting points ranging from 0.5 cm × 0.5 cm–5 cm × 5 cm (see Table 1).

Individual mechanisms of electromagnetic shielding efficiency, such as reflection and absorption, were also studied, but due to the scope of the work, only the overall shielding efficiency will be presented in this chapter. However, it can be summarized that for all the samples studied, the shielding efficiency by reflection prevailed, which is caused by the presence of a relatively large amount of metal fiber (20 %) in the structure of the textile samples.

### 3.1 Electromagnetic SE versus different frequencies and distribution of connecting points of 3D multi-layered weft interlaced woven fabric samples

**Fig. 6:**
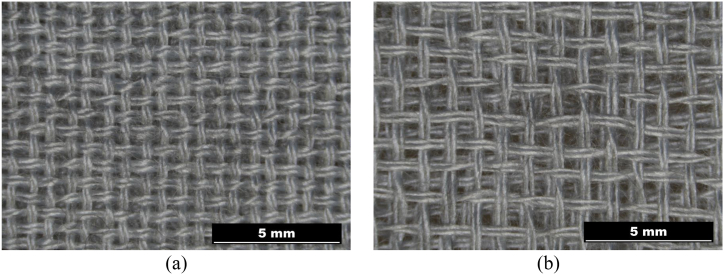
Macroscopic images of real samples: (a) 2-layer 3D woven fabric without connecting points (*sett of ends/picks for one layer 13.*2/cm*/13.*2/cm), (b) 3-layer 3D woven fabric without connecting points (*sett of ends/picks for one layer 8.*8/cm*/8.*8/cm).

**Fig. 7:**
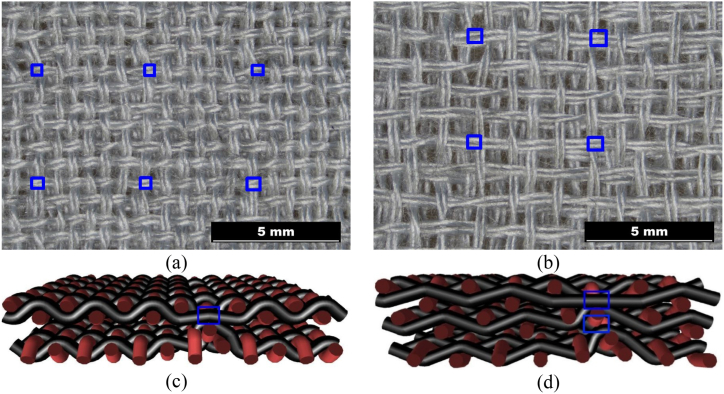
Macroscopic images of real samples: a) 2-layer 3D woven fabric with connecting points (blue mark), b) 3-layer 3D woven fabric with connecting points (blue mark; model of: c) cross-section of 2-layer 3D woven fabric with connecting points and float part (blue mark), d) cross-section of 3-layers 3D woven fabric with connecting points and float part (blue mark).

**Fig. 8:**
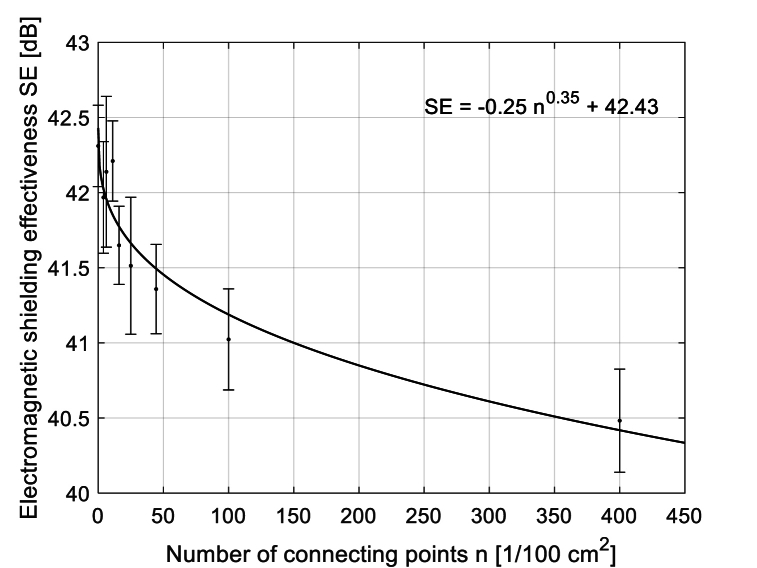
Dependence of SE (*f* = 1.5 GHz) on number of connecting points in 2-layer 3D woven fabric samples, Group 2.

The structure in a plain weave of 3D multilayer woven fabrics is shown in Fig. 6, Fig. 7 (*note: images of woven fabrics were captured by an optical microscope NIKON ECLIPSE E 200, camera NOPTIK PROGRES CT3, using a 2* × *0.6 magnification (2.*65μm*/px, resolution 2048* × *1536*). The dependence of SE on number of connecting points (*n*) for double-layer 3D samples is shown in Fig. 8. This group contains samples having warp and weft density 13.2 yarns/cm and connecting points with a spacing from 0.5 cm to 5 cm, both in the warp and in the weft, which corresponds to the number of joints from 4 to 400 per 100 cm^2^, see Table 1. The same dependence for double-layer and three-layer: two layers are connected only, and three-layer 3D woven fabrics: all three layers are connected, as shown in Fig. 8. These samples have sett of ends/picks for one layer 8.8/cm/8.8/cm. The result of measuring the electromagnetic SE shows decreased SE with increasing connecting points in the 3D multi-layered weft interlaced fabrics. This result is evident for all 2-layer and 3-layer 3D fabric samples. Based on the analysis of the internal geometry of connecting points in 3D multilayer weft interlaced woven fabrics, it is confirmed that the decrease of SE is related to a float part (non-interlacing part of threads) in the connecting point. The float part arises in one layer of the fabric in place of the connection, see Fig. 6. In general, the presence of the float parts decreases the electromagnetic SE. Our previous study's results focused on the weave influence in 2D woven fabrics on electromagnetic SE [12] confirmed that the electromagnetic SE depends on the number of interlacing (crossing) points as well as the float length of the warp and weft threads in woven fabric weave repeat. The number of interlacing (crossing) points between ends and picks increases the electromagnetic SE, and the float parts decreases the electromagnetic SE.

**Fig. 9:**
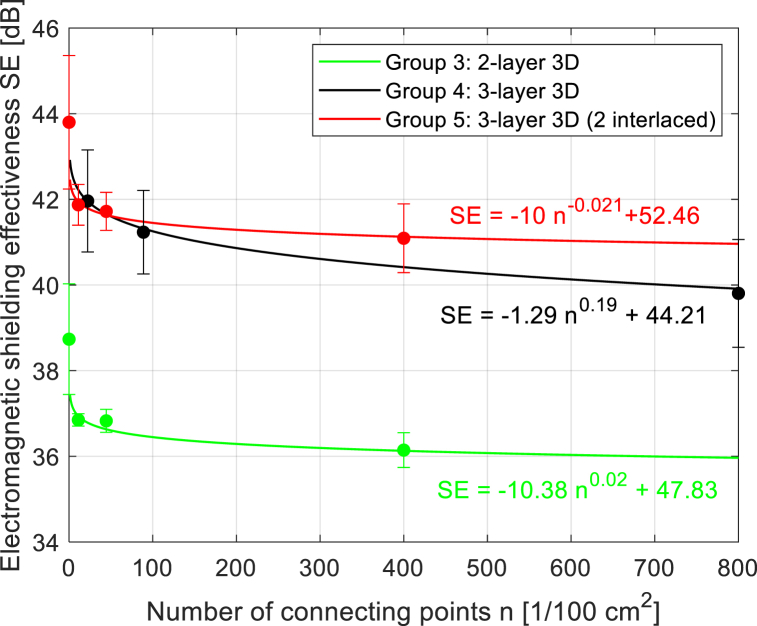
Dependence of SE (*f* = 1.5 GHz) on the number of connecting points in 2-layer 3D fabrics (8.8 threads/cm (Group 3), and 3-layer fabrics (8.8 threads/1 cm (Group 4), and 3-layer fabrics (only 2 layers from 3 are interlaced) (8.8 threads/1 cm (Group 5).

During the examination of Fig. 9, the following can also be observed in addition to the decrease in SE with the increasing number of connecting points in the fabric structure: the highest SE is achieved by three-layer samples without connecting points, which is due to a higher number of layers (higher GSM, higher thickness, higher total warp, and weft density) compared to two-layer ones. Let's compare the samples of Groups 4 and 5. It is apparent that it is more advantageous to use a 3-layer fabric, where only two layers are connected (one remains free), which is again related to the number of connecting points and the presence of floats in the structure. The fewer floats, the higher the SE.

The total length of float parts in connecting points can be estimated based on the frequency of connecting points per defined area. From the internal geometry of the woven fabric cross-section, the length of the float parts is geometrically set to the mean yarn diameter *d* = 0.254 mm which was used for calculation of potential float length in connecting point (n*ote: the yarn diameter is an experimental value.* The diameter is expressed from the area view of the fabric after scanning. *The images of yarn were successfully observed and captured by an optical microscope NIKON ECLIPSE E 200, camera NOPTIK PROGRES CT3, using the 2×0.6 magnification (2.*65μm*/px, resolution 2048x1536). Evaluation of individual images of yarn was made using software of image analyses NIS-Elements. Measurements was repeated 10 times at a different place within the length of the yarn, 95 % CI* (0.249; 0.259)). Table 2 presents the total length of the float parts converted to an area of 100 cm^2^.

**Table 2:** The total lengths of the float parts in connecting points of 2D and 3D multi-layered weft-interlaced woven fabric samples were recalculated to 10 cm^2^.

	Type of fabric	Number of layers	Distribution of connecting points [cm]	Number of connecting points [/100 cm^2^]	Length of float in connecting points [cm/100 cm^2^]
Group 0	2D	1	–	–	0
	2D	1	–	–	0
Group 1	3D	2	0	0	0
			0.5/0.7	285	7.24
		2	0	0	0
			0.5/0.5	400	10.16
		2	0	0	0
			0.5/0.38	526	13.36
		2	0	0	0
			0.5/0.29	690	17.53
Group 2	3D	2	0	0	0
			0.5	400	10.16
			1	100	2.54
			1.5	44.44	1.13
			2	25	0.64
			2.5	16	0.41
			3	11.11	0.28
			4	6.25	0.16
			5	4	0.10
Group 3	3D	2	0	0	0
			0.5	400	10.16
			1.5	44.44	1.13
			3	11.11	0.28
Group 4	3D	3	0	0	0
			0.5	800	20.32
			1.5	88.88	2.26
			3	22.22	0.56
Group 5	3D	3 (2 interlaced only)	0.5	400	10.16
			1.5	44.44	1.13
			3	11.11	0.28

Note: Group 4: 3-layer fabrics – connecting points are created between first + second layer and between second + third layer, which is why the number of connecting points is doubled compared to other 2-layer fabrics where connection is only between the first and second layer.

**Fig. 10:**
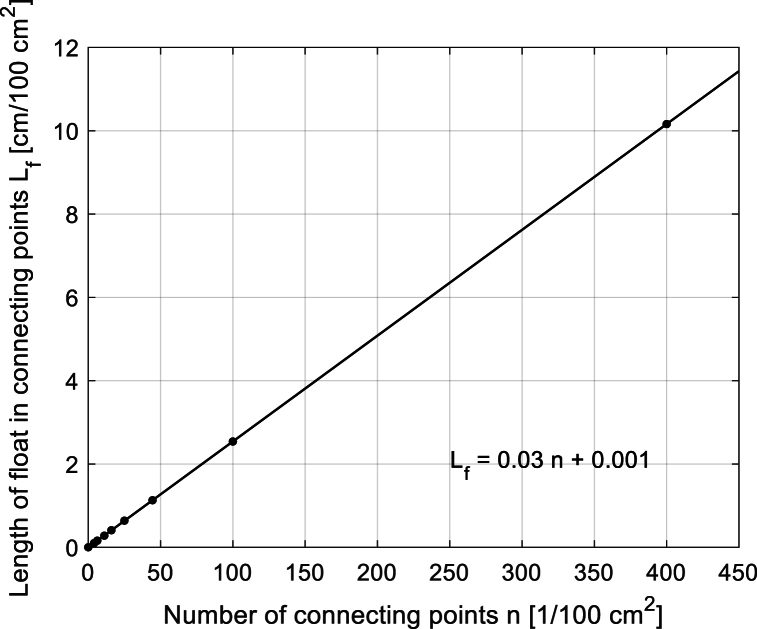
Dependence of the total sum of the floats length on the number of connecting points for sample Group 2.

The dependence of the total sum of the length of the floats is directly proportional to the total number of connecting points, see Fig. 10 given by Table 2.

**Fig. 11:**
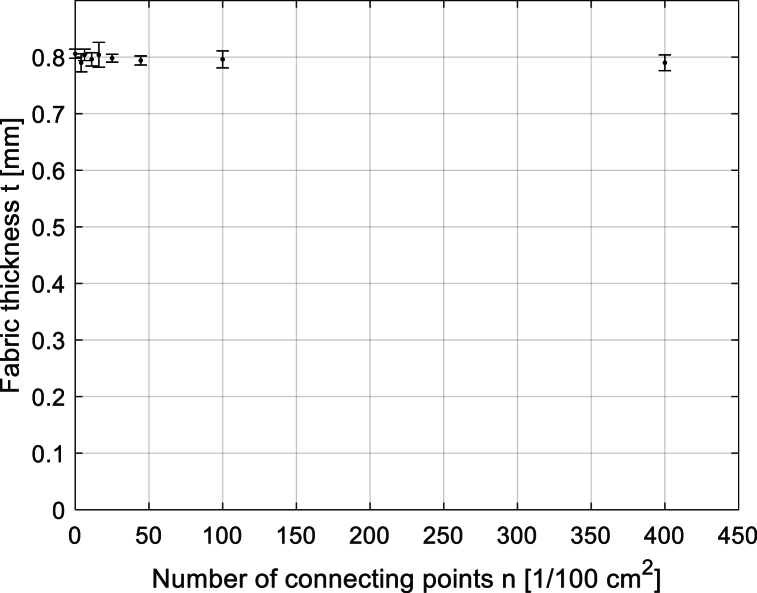
The dependence of the sample thickness on the total number of connecting points for sample Group 2.

**Fig. 12:**
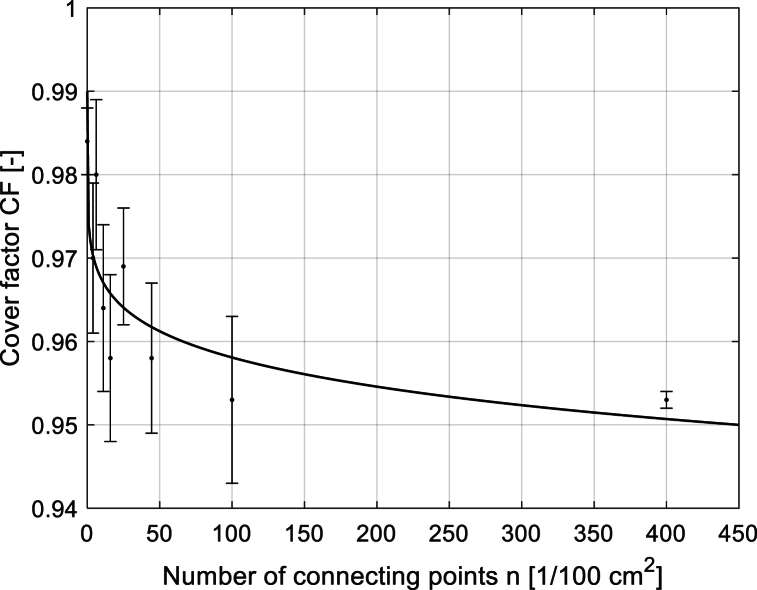
Dependence of the sample CF on the total number of connecting points for sample Group 2.

The influence of the number of connecting points on the sample thickness and sample cover factor was also investigated, and these dependencies for samples of Group 2 are seen in Fig. 11, Fig. 12. Using one-way ANOVA, see Table 3, the *p*-value higher than the significance level (*α* = 0.05) indicates that the mean thicknesses of samples with different connecting points are the same. In other words, the statistically significant effect of the number of connecting points on the sample thickness was not confirmed. On the contrary, using the same statistical method (see ANOVA results in Table 4), it was confirmed that the mean CF of samples with different numbers of connecting points is different. The small *p*-value of 0.00006 indicates that the differences between the CF means of samples with different connecting points are significant. It seems that the higher the number of connecting points in the fabric, the lower its CF is due to the formation of pores (note: pores are given by float in connecting point) in the local cross-linking of yarn connecting the two layers. Similar behavior was also observed in other groups of samples.

**Table 3:** Results table from one-way analysis of variance for thickness.

Source	Sum of squares	Degrees of freedom	Mean square	F	*p*-value
Number of connecting points	0.00143	8	0.00018	0.79	0.62
Error	0.0082	36	0.00023		
Total	0.00963	44

**Table 4:** Results table from one-way analysis of variance for CF.

Source	Sum of squares	Degrees of freedom	Mean square	F	*p*-value
Number of interlacements	0.00966	8	0.00121	4.9	5.85E-05
Error	0.01995	81	0.00025		
Total	0.02962	89			

### 3.2 Electromagnetic SE versus sett of picks of 2-layer 3D multi-layered weft interlaced woven fabric samples

**Fig. 13:**
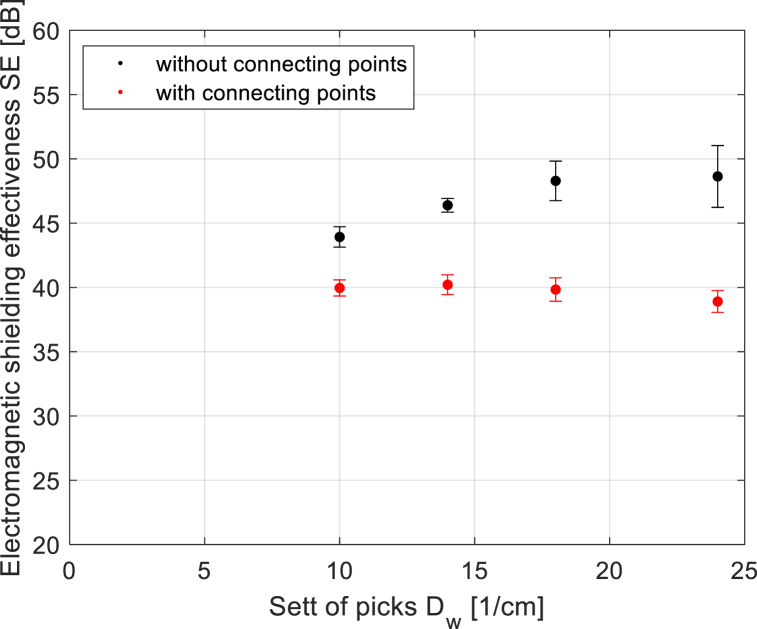
Dependence of SE (*f* = 1.5 GHz) on the sett of picks in 2-layer 3D woven multi-layered fabrics (Group 1 samples). Note: red marks - two-layer 3D woven multi-layered fabrics with connecting points, black marks - two-layer 3D woven multi-layered fabrics without connecting points.

**Fig. 14:**
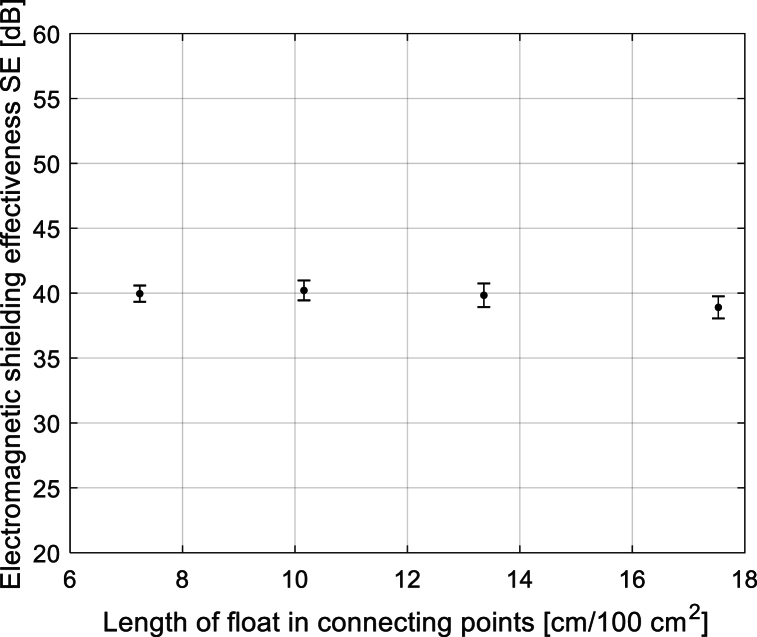
Dependence of SE (*f* = 1.5 GHz) on the total sum of floats length in connecting points of 2-layer 3D woven multi-layered fabrics vith connection points (see Group 1 samples in Table 2).

From the various contributions of authors dealing with the influence of the sett of ends/picks (thread densities) in 2D woven fabrics on SE, it follows that changing the sett of ends/picks in the fabric leads to a change in SE. Increasing the sett of ends/picks of 2D fabric increases the SE. This trend has been confirmed for both 2D and 3D woven multi-layered fabrics without connecting points, both 2-layer and 3-layer. However, this result was not confirmed for 3D woven multi-layered fabrics with connecting points. The SE decreases by increasing the sett of ends/picks in the 3D woven multi-layered fabrics with connecting points. Analysis of this group of fabric samples (Group 1) confirmed that an increase in the sett of ends/picks leads to: a) a change in the position of the connecting points in the fabric and b) increasing the number of connecting points in the area of the 3D fabric. As mentioned above, a float part is created at the connecting point. SE decreases by increasing the length of the float parts in the area of the 3D multi-layered interlaced fabrics, which was confirmed by the results and measurements, see Table 2 and Fig. 13, Fig. 14. Fig. 13 shows an increase of the SE based on increasing sett of picks in 2-layer 3D woven multi-layered fabrics without connecting points, as well as decreasing SE based on increasing sett of picks in 2-layer 3D woven multi-layered fabrics with connecting points. Fig. 14 shows a justification for reducing the SE efficiency based on increasing the total length of the float parts in connecting points.

### 3.3 Electromagnetic SE versus the number of layers of 3D multi-layered woven fabric samples

As already presented in the previous chapter, the number of threads per defined length of the fabric (threads/cm) or per designated area of the fabric (threads/cm^2^) determines the behavior of the fabric from the point of view of electromagnetic SE. The sett of ends/picks and number of layers in 3D multilayer fabrics without connecting points can change the total number of threads in woven fabric. From the analysis of the influence of the number of layers of 2D fabric in a 3D multilayer fabric, it follows that increasing the number of layers increases the SE, see Fig. 12.

Two fabric constructions were included for analysis, one with the sett of ends/picks equal to 8.8/cm and the second fabric construction where the sett of ends/picks is equal to 13.2/cm.

**Fig. 15:**
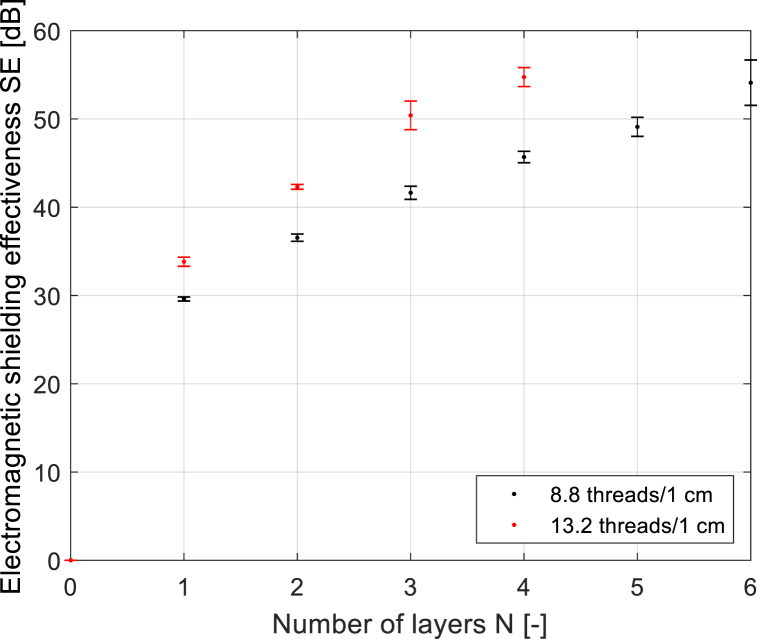
Dependence of SE (*f* = 1.5 GHz) on the number of layers in 3D multilayer fabrics without connecting points (*note: these 3D multilayer fabrics with different number of layers are created by layering of 2D fabrics with a defined number of layers of 2D fabrics, where the layers are not interconnected*).

**Fig. 16:**
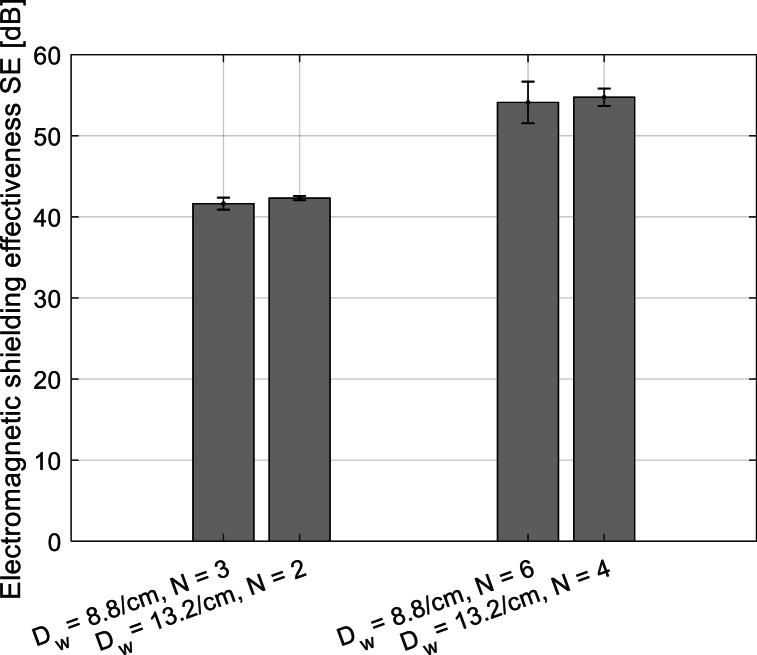
Comparison of 3D multi-layered fabrics with the same SE efficiency (*f* = 1.5 GHz) achieved by the number of layers (N) and sett of ends/picks (*D*_*w*_) 8.8/cm and 13.2/cm in the woven fabric.

From Fig. 15, it can be seen that the same SE is achieved using a different number of layers in combination with the correct sett of ends/picks. In our case, the sett of ends/picks in fabrics is equal 8.8/cm and 13.2/cm. For example, two layers of fabric with sett of ends/picks 13.2/cm provide the same effective thread count as three layers with sett of ends/picks 8.8/cm, causing identical SE. These fabrics with the same SE are compared separately in Fig. 16.

**Fig. 17:**
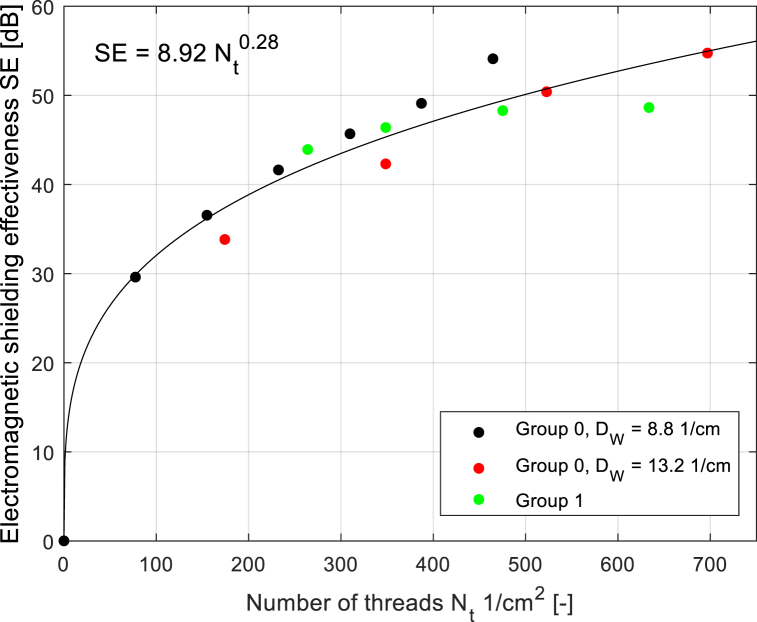
Dependence between SE (*f* = 1.5 GHz) and number of electrically conductive threads N_t_ in the plain weave fabric together with the prediction equation.

Based on the findings described above, it is possible to plot the dependence of SE on a number of threads/cm^2^ (see Fig. 17), which depends on the knowledge of the number of layers and warp and weft sett for the fabrics with plain weave. Two groups of samples were included to this analysis (layered samples from Group 0, two-layer samples from Group 1 without connecting points). The dependence was approximated by the following power function:

(1) $$SE=8.92\;{N_{t}}^{0.28\;}\text{,}$$

where *N*_*t*_ is number of threads per cm^2^, with coefficent of determination *R*^*2*^ = 0.96. SE experimentally evaluated, SE analytically estimated according to Eq. (1), and absolute errors $e_{i}=\left|{SE}_{analytical}-{SE}_{experiment}\right|$ for ten chosen levels of number of threads/cm^2^ (due to limited space, selected data are presented) are shown in Table 5. It si visible that the maximum absolute error is 5.67 dB. To numerically express the difference between the experimental values and the modeled values of SE, mean absolute error (MAE) [35] was computed for all levels of number of threads/cm^2^ (*n* = 15):

(2) $$MAE=\frac{\sum_{i=1}^{n}\left|{SE}_{analytical}-{SE}_{experiment}\right|}{n}=2.05\;\text{dB}$$

**Table 5:** Comparison of SE measured and SE predicted for 10 chosen levels of number of threads.

*N*_*t*_ [1/cm^2^]	77.4	174.2	232.3	264	309.7	348.4	387.2	475.2	633.6	696.9
*SE*_*experiment*_ [dB]	29.6	33.8	41.6	43.9	45.6	42.3	49.1	48.2	48.63	54.7
*SE*_*analytical*_ [dB]	30.1	37.8	41.0	42.5	44.4	45.9	47.3	50.1	54.3	55.77
*error*	0.54	4.00	0.62	1.42	1.23	3.62	1.79	1.81	5.67	1.03

The random error of the coaxial transmission line method, as reported in the standard (see Ref. [28]), is ±5 dB. Therefore, the MAE for the presented approximation is within the range of the random error of the measurement method. Thus, an approximation model can be used to successfully predict SE based on the knowledge of the number of threads per cm^2^.

Using the regression equation, the SE (for *f* = 1.5 GHz) was predicted (with the use of extrapolation and interpolation operations) based on the intended number of threads/cm^2^ (independent variable), see Table 6.

**Table 6:** Predicted electromagnetic shielding effectiveness according to Eq. (1).

*N*_*t*_ [1/cm^2^]	0	50	100	200	300	400	500	600	700	800	900	1000
*SE* [dB]	0	26.7	32.4	39.3	44.0	47.7	50.8	53.5	55.8	58.0	59.9	61.7

## 4 Conclusion

The main objective was to determine whether the interconnection of the fabric structure affects the ability of the 3D fabric to shield electromagnetic fields. Interconnection points are usually advantageously used to improve the mechanical properties of woven structures, especially when using textiles as reinforcements in composites. For this experiment, 2D and 3D woven fabrics were chosen in a plain weave, formed by an electrically conductive hybrid yarn with a fineness of 40 tex containing 20 % staple stainless steel fibers mixed with polyester fibers. During the weaving, the layers in 3D woven fabrics were connected using weft interlacing with different distances of connecting points.

The primary finding is that interlacing woven layers at specific points in a plain weave pattern leads to a statistically significant decrease in overall shielding efficiency. Furthermore, it was found that the influence of the variable “number of interlacing points” (negative effect on SE) is greater than the influence of the variable “sett of ends/picks” (positive effect on SE). This fact is caused by flots, i.e., loose threads at the connecting points, which is consistent with papers that have studied the effect of interlacing within 2D woven textile structures [13,[36], [37], [38]]. The higher the number of connecting points per unit area, the higher the total length of the floats per unit area. A statistically significant decrease of electromagnetic shielding effectiveness given by connecting points has been observed both in two-layer interlaced woven fabrics and in three-layer fabrics, where two or even three layers are connected. From the point of view of achieving the highest possible SE, it appears that layering without bonding in the fabric structure is most advantageous. These findings are in good agreement with theoretical analysis of electromagnetic wave propagation, where it is known that not only the quantity of the conductive component (metal fiber content in yarn, weft/warp density) but also its distribution causing openings (pattern, number of connecting points in multilayer fabrics influencing length of floats and cover factor) have a significant effect on the overall electromagnetics.

Furthermore, it was found that by layering fabrics in plain weave, a higher number of threads per unit area of the fabric can be achieved, while knowledge of the number of electrically conductive threads can be used to predict SE using a simple power relation. The proposed relationship is limited to the given type of thread.

It should be mentioned that it is assumed that the presented textile structures containing stainless steel fibers could be used in standard climatic conditions. In addition, it has been tested that structures containing metal fibers also resist maintenance relatively well, or repeated washing and drying, as seen in already published work [39]. Nevertheless, the electromagnetic shielding efficiency changes of fabrics after long-term use and repeated washing can be taken as the future research direction.

The comparison of internal fabric microstructures reveals statistically significant differences in SE, extending theoretical guidance on woven fabric design by introducing 3D multi-layered interlaced fabrics with controlled SE. The study highlights the need for further research in 3D woven structures to better control SE according to diverse fabric structures. It contributes to both basic and applied research: in basic research, it describes the structure of 3D multi-layered weft interlaced fabrics, presents a model for predicting SE based on thread density, and identifies unexplored fabric morphologies. In applied research, the study suggests applications of designed textile structures as clothing textiles for protective light wear, and as technical light textiles, such as light reinforcement in composite structures or protective light covers in automotive with potential for tubular shielding covers using 3D structures and shuttle weaving techniques.

## CRediT authorship contribution statement

**Brigita Kolcavová Sirková:** Writing – review & editing, Writing – original draft, Validation, Supervision, Methodology, Investigation, Funding acquisition, Formal analysis, Conceptualization. **Veronika Tunáková:** Writing – review & editing, Writing – original draft, Methodology, Investigation, Data curation. **Maros Tunák:** Writing – original draft, Investigation, Formal analysis, Data curation. **Karol Jezik:** Visualization, Investigation.

## Data availability

Data will be made available on request.

## Funding

The author(s) disclosed receipt of the following financial support for the research, authorship, and/or publication of this article: This work was supported by the Ministry of Education, Youth and Sports of the Czech Republic and the European Union—European Structural and Investment Funds in the Frames of Operational Program Research, Development and Education—Project Hybrid Materials for Hierarchical Structures [HyHi, Reg. No. CZ.02.1.01/0.0/0.0/16_019/0000843].

## Declaration of competing interest

The authors declare the following financial interests/personal relationships which may be considered as potential competing interests: Brigita Kolcavova Sirkova reports financial support was provided by 10.13039/501100005280Technical University of Liberec Faculty of Textile Engineering. If there are other authors, they declare that they have no known competing financial interests or personal relationships that could have appeared to influence the work reported in this paper.
